# Arthrofibrosis after TKA - Influence factors on the absolute flexion and gain in flexion after manipulation under anaesthesia

**DOI:** 10.1186/1471-2474-12-184

**Published:** 2011-08-12

**Authors:** Ingmar Ipach, Falk Mittag, Julia Lahrmann, Beate Kunze, Torsten Kluba

**Affiliations:** 1 Department of Orthopaedic surgery, University Hospital of Tuebingen, Hoppe-Seyler-Str. 3, 72076 Tuebingen, Germany

## Abstract

**Background:**

Stiffness with decreased range of motion (ROM) has been described as a frustrating complication after TKA. If all methods of physiotherapeutic treatment have been exhausted trying to develop ROM, manipulation under anaesthesia (MUA) can be discussed. The aim of the present study was to show the effect of MUA and to determine the influence of BMI, number of previous surgical procedures, pre-MUA ROM and timing of MUA for the results after MUA in regard to absolute flexion and gain in flexion.

**Methods:**

858 patients underwent TKA at our institution between 2004 and 2009. 39 of these patients underwent MUA because of postoperative knee stiffness. The data were retrospective analysed for the influence of BMI, pre-MUA flexion (</≥ 70°), timing of MUA (>/≤ 30 days after TKA) and number of previous surgery on the results after MUA (absolute Flexion/gain in flexion).

**Results:**

The prevalence for stiffness after TKA was 4.54%. There was a statistically significant improvement in flexion not only directly after MUA but also 6 weeks after MUA. Patients with two or more previous operations before TKA showed statistically significant worse results six weeks after MUA in absolute flexion and gain in flexion

(p = 0.039) than patients with one or two previous operations. No statistical significance in absolute flexion (p = 0.655) and gain in flexion (p = 0.328) after MUA between "early" and "late" was detected. The stiffer knees with a flexion below 70° showed significantly worse results (p = 0.044) in absolute flexion six weeks after MUA, but they also had statistical statistically better results with regard to gain in flexion (p ≤ 0.001).

**Conclusion:**

MUA is a good instrument for improving ROM after TKA. The time between TKA and MUA seems less important, so different types of physiotherapeutic treatment could be tried before the procedure is started. MUA in patients with many previous operations and a flexion of less than 70° before MUA is not as effective as in other patients, but they also benefit from MUA.

## Background

Most people who receive a total knee arthroplasty (TKA) because of late stage osteoarthritis experience a great benefit with regard to mobility and quality of life. Although x-rays show a perfect position of the knee implant, arthrofibrosis and the associated reduced range of motion (ROM) can be a frustrating complication for both the patient and the surgeon. Laubenthal et al. showed that patients require 67° of knee flexion during the swing phase of gait, 83° are required to ascend stairs, between 90° and 100° of flexion are required to descend stairs. To stand up from a normal sized chair, people need a knee flexion of about 93° [1]. The aim after TKA is therefore a ROM of about 90°-100° to ensure that there are no problems in daily life. Vigorous rehabilitation after TKA is important to reach this value.

However, the reasons for losing an adequate ROM are manifold with factors including the patient's own healing process and formation of fibrous scar tissue [2-4].

If all methods of physiotherapeutic treatment have been exhausted trying to develop this ROM, manipulation under anaesthesia (MUA) can be discussed.

Previous studies have already shown lasting gain in flexion mobility following manipulation under anaesthesia to treat inadequate ROM after TKA [2,3].

Although manipulation under anaesthesia is a non-invasive procedure to increase ROM, it may cause complications such as femoral supracondylar fracture, rupture of the patellar ligament, wound dehiscence and haemarthrosis [4]. Therefore the risk-benefit ratio should be carefully considered.

The aim of the present study was to determine prevalence-data for the development of stiffness after primary TKA and to show the efficiency of MUA for the treatment of postoperative lack of ROM in regard to total flexion and gain of flexion in the early and late postoperative period.

Furthermore we tried to determine causative factors for the results after MUA. Therefore the study wanted to prove the influence of BMI, time since primary TKA (>/≤ 30 days), number of previous surgical procedures and flexion before MUA on the results (total flexion/gain of flexion) 6 weeks after MUA.

## Methods

Data files from patients who underwent a manipulation under anaesthesia (MUA) to treat arthrofibrosis after TKA (Genesis II^® ^Smith&Nephew) were retrospectively analysed. If a flexion of more than 90° after TKA was not achieved, they were entitled as being stiff. All patients with "stiff knees" underwent MUA during the inclusion period between August 1, 2004 to July 31, 2009.

The in-house rehabilitation protocol after TKA was the same for all patients during the 5-year-period. All patients received a 3-1 femoral block and intravenous anaesthesia for pain management, mobilisation with walking sticks on the first postoperative day, active and passive flexion exercises with the physiotherapist and continuous passive motion twice a day for 30 min. The 3-1 femoral block was removed on day 3 after surgery.

All patients had to achieve a ROM of >90° before discharge from hospital. If a ROM of >90° was not achieved, the patient's length of hospital stay was prolonged by up to 14 days. If a ROM of 90° was still not achieved after this time, the patients were encouraged to undergo immediate MUA. The ROM was documented on a daily basis by an independent physiotherapist using a goniometer. The measurements took place before arthrotomy/MUA, every day during the postoperative in-patient period and 6 weeks postoperatively in the outpatient clinic follow-up appointment.

To determine the effect of time between primary TKA and MUA we analysed two different groups (early/late manipulation group).

All patients with an MUA before the 30th day after surgery were included in the "early manipulation group". The "late manipulation group" were those patients who achieved a flexion of 90° until discharge but a decrease in ROM was seen after the 30th day after surgery.

The numbers of previous surgical procedures (arthrotomy and arthroscopy) before MUA were recorded by asking the patient and by analysing the individual medical records. To determine the effect we split the patients in different groups (one previous operation/two previous surgeries/>2 previous operations).

The metric BMI (body mass index) was evaluated with the common formula [weight in kilograms/height in meters^2^]. Three groups (underweight/normal/overweight) were compared in regard to results after MUA. A BMI < 18.5 was defined "underweight", 18.5 - 24.9 "normal", 25 - 29.9 "overweight" and >30 "obese".

In another step, all patients were divided into two groups with a flexion of > 70° before TKA and < 70° before TKA. These two groups were also analysed for the effect of MUA (total flexion/gain of flexion).

### Manipulation protocol

Before induction of a general anaesthesia, all patients received a 3-1 femoral block. After induction of a general anaesthesia the hip was flexed to 90°. After this the knee-joint was gently flexed and extended until palpable lysis of adhesions was completed and a ROM of 120°-130° was achieved. The knee was held in this position for several seconds. Then the knee was flexed and extended into the maximum position several times. After manipulation, x-rays of the knee joint were taken to rule out an iatrogenic fracture.

As soon as the patients left the recovery room, the physiotherapist showed them active-assisted ROM-exercises. During the following days the patients were treated by the therapist with active and passive exercises and continuous passive motion. The ROM was documented daily and the femoral-block was stopped and removed on day 3. Cryotherapy with ice-packs placed on the knee was used every day at the discretion of the physiotherapy staff.

### Statistics

All data were organized with "Microsoft Office Excel" and analyzed with "PASW SPSS 17".

A p-value of < 0.05 was considered significant.

A Kolmogrov-Smirnov-test and a quantile-quantile-plot were used for testing group variance and normal distribution.

Two-sample t-tests were used for side-by-side comparisons of preoperative flexion and flexion before MUA; flexion before MUA and flexion after MUA; flexion before MUA and flexion 6 weeks after MUA; flexion after MUA and flexion 6 weeks after MUA.

A one-factorial variance analysis (ANOVA) and the Turkey-Kramer-test were used to determine factors influencing the outcome after MUA.

The study was sufficiently powered to detect an effect size greater than 0.92 with the two-sample t-test and a 0.05 level of significance.

## Results

858 TKAs were performed at our institution during the time between August 1, 2004 and July 31, 2009.

The prevalence for stiffness after TKA was 4.54%. The prevalence for stiffness in patients with one previous operation before MUA was 1.98%, with two previous operations 1.63% and 0.93% in patients with more than two previous operations. The average age of patients who underwent MUA was 64.15° ± 11.19° (range 42-82°) years. 32 (82.1%) of them were female and 7 (17.9%) were male Table 1.

**Table 1:** Demographic data of all patients who received MUA and after separation in different observation groups.

	sex	n =	age in years	Total flexion	Gain in flexion
All patients	f: 32 m: 7	39	64.15 ± 11.19	94.46° ± 14.1 (60° - 125°)	26.5 ± 19.5 (0°-80°)

BMI					
1. < 18.5	f: - m: -	-			

2. 18.5 - 24.9	f:4 m:1	5	58.44 ± 8.79	98.9° ± 10.3° (80° - 110°)	34.4° ± 17.9 (15° - 70°)

3. 25 - 29.9	f:13 m:3	16	64.56 ± 5.79	106.3° ± 10.6° (90° - 130°)	36° ± 13.6° (15° - 70°)

4. >30	f:15 m:3	18	65.37 ± 7.98	97.9° ± 9.6 (75° - 120°)	31.5° ± 18.4° (0° - 80°)

Flexion before MUA					
1. ≥70°	f: 13 m: 3	16	63.45 ± 8.65	106.2° ± 11° (90° - 130°)	24.7° ± 10.6° (0° - 80°)

2. <70°	f: 19 m: 4	23	64.63 ± 6.43	100.0° ± 7.68° (90° - 120°)	39.68° ± 17,221 (10° - 80°)

Previous surgeries					
1. one	f: m:	17	62.11 ± 7.43	103.9° ± 10.22° (90°-130°)	35.56° ± 17.89 (0°-70°)

2. two	f: m:	14	64.53 ± 6.52	102.4 ± 11.06° (75°-120°)	35.22° ± 15.77° (15°-80°)

3. >two	f: m:	8	67.71 ± 5.34	94.1° ± 7.7° (80°-110°)	27.3° ± 15.7° (10°-60°)

Time between TKA and MUA					
1. >30 days	f: m:	11	65.34 ± 6.43	103.7° ± 9.8° (90° - 130°)	28.9° ± 20.9° (-5 - 70)

2. <30 days	f: m:	28	63,65 ± 5.32	98.9° ± 8.2° (90 - 110°)	18.3° ± 16.4° (-10° - 40°)

The duration of stay in hospital after TKA was 13.49 days ± 4.77 (range 6-28 days).The length of stay in hospital after MUA was 9.08 days ± 4.3 (range 3-24 days). The mean BMI of all patients who underwent MUA was 30.12 kg/m^2 ^± 5.17 (range 21.51 - 41.03 kg/m^2^). 5 (12.8%) patients were of normal weight, 16 (41%) were overweight and 18 (46.2%) were obese. None was underweight.

The mean flexion before TKA was 104.87° ± 17.23° (range 70 - 140°)

Manipulation took place at a mean of 75.7 ± 62.6 days (range 12-218 days) after TKA.

**Figure 1:**
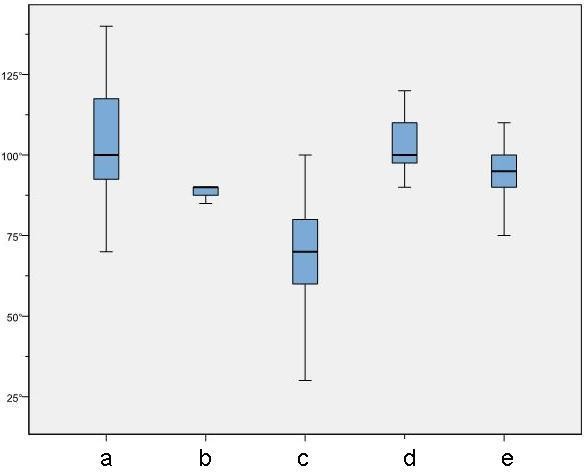
**Box-plot-diagram (Min, 25% Quantil, Median, 75% Quantil, Max) showing flexion to different points of time**. a) before TKA; b) after TKA; c) before MUA; d) after MUA; e) six weeks after MUA. A statistical significant change in flexion was seen between the different points of time (p ≤ 0.001).

The development and changing in ROM to the different points of time are shown in Figure 1.

17 patients (43.6%) developed arthrofibrosis after primary TKA, 14 patients (35.9%) developed arthrofibrosis with the TKA as second procedure and 8 patients (20.5%) developed arthrofibrosis after two or more operations before the TKA was implanted.

The mean flexion six weeks after MUA in patients who developed arthrofibrosis after primary TKA was 103.9° ± 10.22° (90 - 130°) with a gain in flexion compared to pre-MUA values of 35.56° ± 17.89 (0 - 70°). The mean flexion in patients with TKA as second procedure was 102.4° ± 11.06° (75 - 120°) with a gain in flexion of 35.22° ± 15.77° (15 - 80°). Patients with two or more previous operations before TKA showed statistically significant worse results six weeks after MUA in absolute flexion and gain in flexion (p = 0.039) than patients with one or two previous operations. The mean flexion in this group six weeks after MUA was 94.1° ± 7.7° (80 - 110°) with a gain in flexion of 27.3° ± 15.7° (10 - 60°). No significant differences were detected between patients after primary TKA and two previous operations.

In 11 cases (28.20%), MUA took place in the first 30 days after TKA, in 28 cases (71.80%) after 30 days. The mean flexion in the "early" group six weeks after MUA was 98.9° ± 8.2° (90 - 110°) with a gain in flexion of 18.3° ± 16.4° (-10 - 40°). The mean flexion in the "late" group six weeks after MUA was 103.7° ± 9.8° (90 - 130°) with a gain in flexion compared to pre-MUA of 28.9° ± 20.9° (-5 - 70°). Considering the data no statistical significance in absolute flexion (p = 0.655) and gain in flexion (p = 0.328) after MUA between "early" and "late" was detected.

The mean flexion in the group with a BMI between 18.5-24.99 kg/m2 was 98.9° ± 10.3° (80 - 110°) six weeks after MUA with a gain in flexion of 34.4° ± 17.9° (15 - 70°), the mean flexion in the group with a BMI between 25-29.99 kg/m2 was 106.3° ± 10.6° (90 - 130°) with a gain in flexion of 36° ± 13.6° (15 - 70°) and in the group with a BMI ≥ 30 kg/m2 the mean flexion six weeks after MUA was 97.9° ± 9.6 (75 - 120°) with a gain in flexion of 31.5° ± 18.4° (0 - 80°). No statistically significant influence of BMI was seen between the different groups in absolute flexion (p = 0.334) and gain in flexion (p = 0.665)

23 patients (59%) had a flexion of less than 70° before MUA, 16 patients (41%) had a flexion greater than 70° before MUA.

The mean flexion in group I (< 70° before MUA) six weeks after MUA was 100.0° ± 7.68° (90 - 120°) with a gain in flexion of 39.68° ± 17,221 (10 - 80°). In group II (≥ 70° before MUA) the mean flexion after MUA was 106.2° ± 11° (90 - 130°) with a gain in flexion of 24.7° ± 10.6° (0 - 80°).

The stiffer knees with a flexion below 70° showed significantly worse results (p = 0.044) in absolute flexion six weeks after MUA, but they also had statistically significant better results regard to gain in flexion (p ≤ 0.001).

## Discussion

Arthrofibrosis of the knee joint leads to a significant decrease in the range of motion (ROM) after TKA and might be a frustrating complication for both surgeon and patient. If all kinds of physiotherapeutic treatment are exhausted and an invasive arthrolysis should be avoided, a closed manipulation under anaesthesia could be started to increase ROM. As there are also some risks in this procedure, a risk-benefit analysis should be performed. We defined the word "stiffness" as a flexion of less than 90° after primary TKA, which is consistent with the definition of previous studies [5-9]. Stiffness after primary TKA has a reported prevalence of 2%-13% [5-9]. In the present study, the prevalence of stiffness after TKA was 4.54% which corresponds with the prevalence published by other authors [10]. The reasons why stiffness develops are poorly understood and are described as being multifaceted [2,3]. In the present study, all TKAs were of an appropriate size and were not malpositioned. No reasons for stiffness other than contracture of the soft tissue were seen.

In our knowledge the present study is first focusing on both, absolute flexion and gain in flexion, after MUA.

Our results indicate that MUA results in significant improvement in flexion, which is consistent with other studies [11,12]. Six weeks after MUA a statistically significant decrease in flexion was measured at the follow-up examinations. A reason for this decrease might be a lower level of physiotherapy after leaving the hospital or a new formation of scar tissue. Otherwise there was still a significant mean improvement in flexion after MUA of about 20°. This is also consistent with previous studies, where a steady decrease has been seen [2]. In our opinion, all patients should receive intensive physiotherapeutic training after leaving hospital - including continuous passive motion - to maintain the ROM achieved after MUA.

In this work a cut off of 30 days was chosen for definition "early" and "late" MUA. Despite the different definition of "early" and "late" MUA our findings are consistent with other studies [3,5,7] as a statistically significant increase in flexion was detected in the early and late MUA group.

Some authors have already shown that there is significant increase in flexion not only for early MUA (<90 days) but also for late MUA (>90 days) but they also showed that there was a significant difference in gain of ROM between early and late MUA and they postulated that an "early" MUA leads to a greater gain of ROM [3,5,7]. In the present study there was no statistically significant gain of flexion in the "early" MUA group. One possible explanation for these finding might be a type 2 error because of a too small sample size.

No other study up to now has shown the influence of previous surgery on the outcome after MUA. To our knowledge, the influence of previous operations on the results after MUA is mentioned [8], but no study really focuses on this topic. The improvement of flexion after MUA in knees with more than two previous operations was statistically significantly worse than in knees with one or two previous surgical procedures. The influence of pre-MUA/preoperative flexion on the outcome after MUA has been controversially discussed. Deluga et al. showed no influence of the preoperative flexion on the results after MUA [5]. On the other hand, previous results by Richard et al. and Kim et al. showed an influence of preoperative flexion on the results after MUA [3,10]. In the present study the results by Richard and Kim could be confirmed, as significantly lower results after MUA in regard to total flexion were found in patients with a flexion of less than 70° before TKA but these patients also benefit (gain in flexion) from MUA.

Fischer et al. [13] implied that there is a significant influence of BMI on the outcome after MUA. Our results could not support these findings as no statistically significant difference was seen between different BMI in absolute flexion after MUA and gain of flexion.

**Figure 2:**
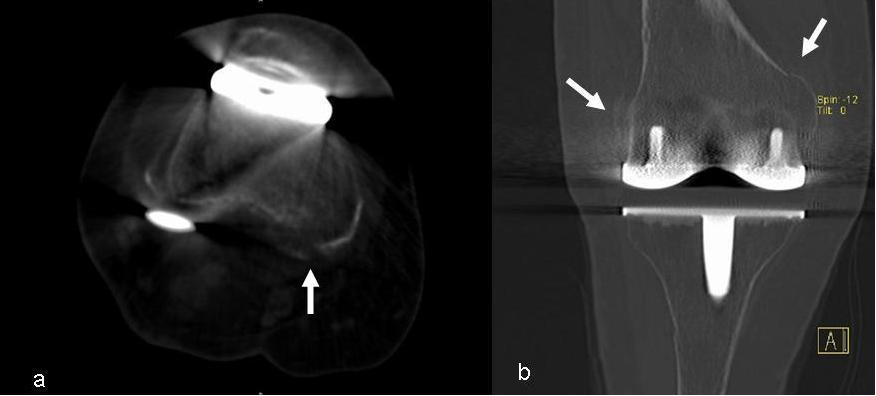
**CT-scan of the right knee-joint**. The fracture line of the periprosthetic fracture is marked by white arrows on an axial view (a) and coronar view (b).

**Figure 3:**
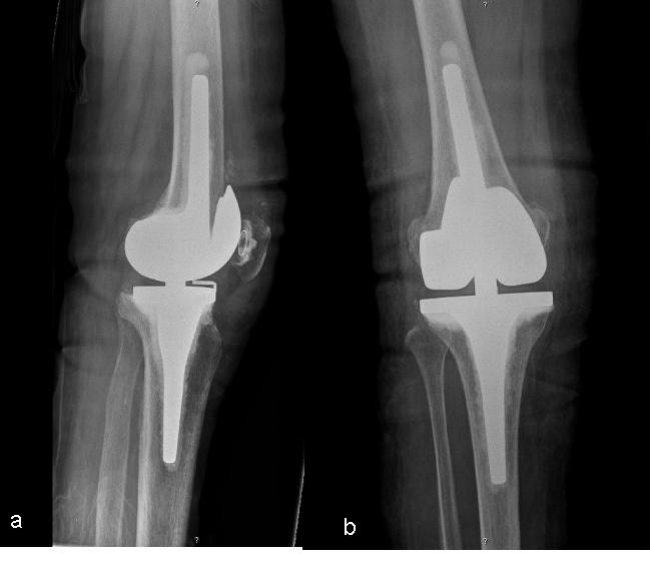
**X-ray showing the axial-guided revision TKA (**Endoplus®**) on a lateral view (a) and a.p. view (b)**.

During the study period there was only one complication. A female patient with osteoporosis developed a supracondylar fracture during MUA. In this case, a change to a femoral revision implant was necessary (Figure 2a/b & Figure 3a/b).

We note several limitations to the study. Firstly, the sample (39 patients) might be too small for any meaningful analysis of "outcome predictors" using a multivariate analysis or a serial univariate test. On the other hand the samples in former studies were even smaller [3,5,7].

Other potential predictors e.g. TKA surgeon, local bleeding, MUA-manipulator were not analyzed but might be of interest. A follow up of six weeks might be too short to find a lasting conclusion, it would be of interest to see the results one or two years after MUA. Follow-up studies should therefore focus on these topics.

## Conclusion

MUA is good instrument for improving ROM after TKA. The time between TKA and MUA seems less important, so all kinds of physiotherapeutic treatment could be tried before the procedure is started. MUA in patients with many previous operations and a flexion of less than 70° before MUA is not as effective as in other patients, but they also benefit from MUA.

## Competing interests

The authors declare that they have no competing interests.

## Authors' contributions

Authors' contributions: II participated in the design of the study, statistical analysis and drafted the manuscript. FM participated in the design of the study and performed the statistical analysis. JL carried out the patient data and performed statistical analysis, BK performed the manipulation under anaesthesia and collected data. TK conceived of the study, and participated in its design and coordination. All authors read and approved the final manuscript.

## Pre-publication history

The pre-publication history for this paper can be accessed here:

http://www.biomedcentral.com/1471-2474/12/184/prepub
